# Unique Static Magnetic and Dynamic Electromagnetic Behaviors in Titanium Nitride/Carbon Composites Driven by Defect Engineering

**DOI:** 10.1038/srep18927

**Published:** 2016-01-07

**Authors:** Chunhong Gong, Hongjie Meng, Xiaowei Zhao, Xuefeng Zhang, Laigui Yu, Jingwei Zhang, Zhijun Zhang

**Affiliations:** 1 College of Chemistry and Chemical Engineering, Henan University, Kaifeng 475004, P. R.China; 2 Engineering Research Center for Nanomaterials, Henan University, Kaifeng 475004, P. R.China; 3 Key Laboratory for Anisotropy and Texture of Materials, Northeastern University, Shenyang 110819, P. R. China

## Abstract

Recently, the defect-induced static magnetic behaviours of nanomaterials have been a cutting-edge issue in diluted magnetic semiconductor materials. However, the dynamic magnetic properties of nanomaterials are commonly ignored if their bulk counterparts are non-magnetic. In the present research, titanium nitride-carbon (TiN/C) nanocomposites were found to exhibit both static and dynamic magnetic properties that vary in the opposite trend. Moreover, novel unconventional electromagnetic resonance behaviour was demonstrated in TiN/C systems, and their permeability and permittivity show similar trend. This is challenging for the traditional understanding of electromagnetism and makes it possible to achieve an appropriate balance between the permeability and permittivity simultaneously in a simple system. Hopefully, the results could provide some valuable clues to revealing the magnetism and electromagnetism of nanostructures.

Nanomaterials are drawing increased attention in recent years, since they usually possess many unique physical properties (mechanical, optical, electrical, magnetic properties and so on) when compared with their bulk counterparts^1^,^2^,^3^,^4^. Due to the high surface to volume ratio of nanostructured materials, various defects (e.g., vacancies, dislocations and dangling bonds) are usually inevitable, which would affect the physical properties of nanomaterials in unexpected way^5^. For example, since Coey *et al.* reported the unexpected *d*^0^ magnetism in a dielectric oxide in 2004, the defect-induced static magnetic properties of nanomaterials have been a cutting-edge issue in diluted magnetic semiconductors (DMSs) materials^6^,^7^. The unique magnetism of nanomaterials is attributed to a variety of structural defects, such as cation vacancies, oxygen vacancies and structural inhomogeneity, as verified by numerous studies on highly defective dilute magnetic oxides and nitrides^7^,^8^,^9^,^10^,^11^,^12^,^13^.

Recently, serious electromagnetic interference has been driving considerable efforts on the development of advanced microwave absorbing material (MAM). The microwave absorption properties are evaluated with the complex permeability (*μ*_r_ = *μ*_r_′ + *iμ*_r_″) and permittivity (*ε*_r_ = *ε*_r_′ + *iε*_r_″). The real part is related to the stored electrical and magnetic energy within the medium while the imaginary part is related to the dissipation of electrical and magnetic energy, and their ratios, the dielectric (tan *δ*_E_ = ε_r_″/ε_r_′) and magnetic dissipation factors (tan *δ*_M_ = μ_r_″/μ_r_′) provide a measure of how much power is lost versus how much is stored in a material^14^,^15^. Ideal MAM should possess not only strong electromagnetic loss ability but also a proper complementariness between the complex permeability and permittivity, so-called impedance match. Up to now, some significant breakthroughs have been achieved by integrating magnetic and dielectric components together^16^,^17^, or turning the inactive MAM into an exciting MAM^18^,^19^. However, few are currently available about the extraordinary electromagnetic behavior of nanostructures upon encountering with dynamic electromagnetic wave^20^. Especially, based on the classical relationship between static magnetic properties and dynamic permeability, it is accepted that only conventional magnetic compositions (such as Fe, Co, Ni, magnetic alloys and ferrites) are considered as the origin of the permeability behavior, and the microwave magnetic loss of nanomaterials is not expected if their bulk counterparts are intrinsically non-magnetic or weak magnetic and the dissipation power is expectedly to be solely determined by dielectric loss^20^,^21^,^22^,^23^. In our very recent researches, we have found that though bulk titanium nitride (TiN) is non-magnetic, TiN nanostructures obtained with the assistance of defect engineering possess both evident static ferromagnetic and dynamic permeability properties^24^,^25^,^26^; and we preliminarily suppose that the composition and microstructure defects have profound effects on both the magnetic and electromagnetic properties. These results remind us to investigate whether there is any relationship between the defect-induced static and dynamic magnetic properties and whether there is any similar mechanism between the permeability and permittivity behaviors of nanomaterials, which remains untouched up to now.

Bearing those perspectives in mind, in the present research we adopt titanium nitride-carbon (TiN/C) nanocomposite as a simple example to investigate the magnetic and electromagnetic behaviours of nanostructured materials. TiN/C nanocomposite is focused on, because it is easy to create the non-stoichiometric solid solutions in the host materials (TiN) by replacing N with heteroatomic substitutions (C) of the similar atomic radius, thereby resulting in more defects. Systematic studies in relation to the composition, structure and defects were conducted, with the hope to acquire some insights into the magnetism and electromagnetism of nanostructured materials and achieve ideal absorption.

## Results

**Figure 1:**
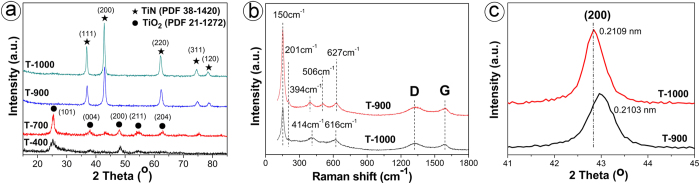
XRD patterns of T-400, T-700, T-900, and T-1000 (**a**), Raman spectra (**b**) and magnified XRD patterns at low 2*θ* (**c**) of T-900 and T-1000.

Figure 1a shows the X-ray diffraction (XRD) patterns of as-obtained samples. It can be seen that only tetragonal anatase TiO_2_ is obtained at 400 °C, and rutile TiO_2_ coexistent with TiN powder is obtained at 700 °C. As the nitriding temperature rises to 900 °C and above, only pure face centered cubic TiN nanoparticles are obtained. As to the Raman spectrum of TiN, it has been evidenced that the scattering in the acoustic range is mainly determined by the vibrations of the heavy metal (Ti) ion vacancies (typically 150–350 cm^−1^) and light element (N) ion vacancies (typically 400–650 cm^−1^)^27^. As shown in Fig. 1b, the Raman peaks of T-900 correspond to anatase TiO_2_ but not TiN. The Raman features at 150 cm^−1^, 394 cm^−1^, 506 cm^−1^ and 627 cm^−1^ are attributed to the Raman-active modes of crystalline anatase TiO_2_ with symmetries of *E*_g_, *B*_1*g*_, *A*_1*g*_ and *E*_*g*_, respectively^28^. Here, it should be noted that the Raman scattering signals of anatase TiO_2_ are very strong, and there are some overlapped signals between anatase TiO_2_ and TiN. This is consistent with what is reported in Ref. 29; that is, even though there is a small amount of anatase TiO_2_ in the as-synthesized TiN samples, the O-Ti-N and Ti-N bonds do not contribute any new Raman band. In other words, though anatase TiO_2_ in sample T-900 could not be detected by XRD, there is still some residual O, due to inadequate degree of nitriding. Differing from T-900, sample T-1000 shows the basic Raman scattering signals of TiN and some typical characteristic peaks of anatase TiO_2_ (for example, the peak at 506 cm^−1^) disappear totally^28^, which means that more complete nitriding reaction occurs at 1000 °C^24^. Besides, both T-900 and T-1000 show D and G peaks of carbon at about 1333 cm^−1^ and 1590 cm^−1^, respectively, which directly proves that they contain carbon. The magnified XRD patterns of as-prepared T-900 and T-1000 are shown in Fig. 1c. It can be seen that the XRD peaks of T-1000 shift towards lower 2*θ* as compared with those of T-900, which means that T-1000 exhibits an increased lattice constant. This well corresponds to relevant mean plane (200) distance of standard TiN (JCPDS card: 38–1420; 0.2121 nm) as well as that of T-900 (0.2103 nm) and T-1000 (0.2109 nm). In combination with corresponding Raman scattering signals, we can reasonably speculate that the smaller lattice constants of T-900 and T-1000 as compared with those of the standard TiN might be attributed to the presence of substitutial oxygen atoms. Increasing nitriding temperature might result in a higher content of N to promote the occupation of more O positions by N atom and to form near-stoichiometric TiN, thereby leading to the increment of the lattice constant of TiN.

**Figure 2:**
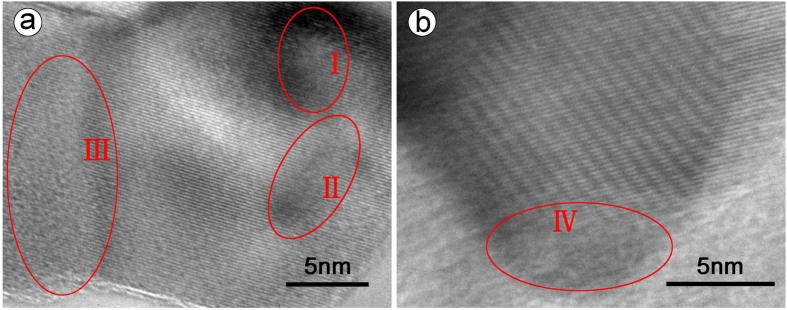
HRTEM images of T-900 (**a**) and T-1000 (**b**).

The transmission electron microscopic (TEM) images of the as-obtained samples are shown in supplementary Fig. S1. It can be seen that both T-900 and T-1000 appear as nanoparticles with an average size of about 20 nm. Figure 2 shows the high-resolution TEM (HRTEM) images taken randomly on sample T-900, which provides evidences to the presence of defects in as-produced TiN powders (such as lattice defects and dislocations in regions “I” and “II”). Considering that the TiN particles obtained under similar conditions without carbon exhibit good crystallinity in the grains and at the grain boundaries (shown in supplementary Fig. S1 in ref. 26), we could infer that the dissolving of a great deal of C and some residual O in association with inadequate nitriding are responsible for the generation of the defects in T-900, because C, N and O can exist as non-stoichiometric solid solution in TiN crystal. In comparison, T-1000 has a well-defined crystalline structure, which indicates that calcining NTA precursor at 1000 °C can well transform it into TiN crystal with good crystallinity. Moreover, the amorphous regions “III” and “IV” at the grain boundaries of T-900 and T-1000 might be assigned to the amorphous carbon and/or the amorphous TiN.

**Figure 3:**
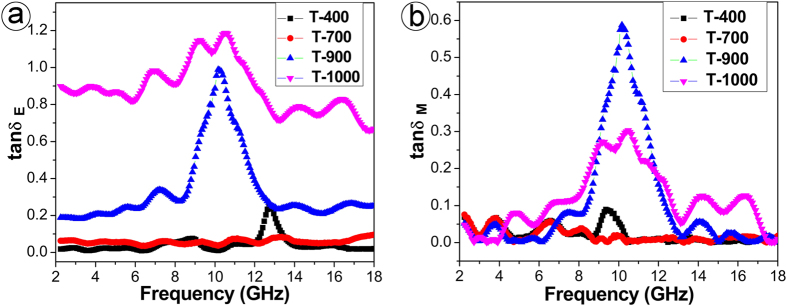
The frequency dependence of tan *δ*_E_ and tan *δ*_M_ of the paraffin composites filled with T-400, T-700, T-900 and T-1000.

The frequency dependencies of the complex relative permittivity and permeability are shown in supplementary Fig. S2. It can be seen that samples T-400 and T-700 have very small *ε*_r_′ and *ε*_r_″ that remain almost constant of 0.5 and 0 in the whole frequency range of 2 ~ 18 GHz. Meanwhile, their *μ*_r_΄ and *μ*_r_″ values are nearly 1.0 and 0 in the whole frequency range. Therefore, their tan *δ*_E_ and tan *δ*_M_ values are ignorable at gigahertz frequency, as shown in Fig. 3. Differing from T-400 and T-700, both T-900 and T-1000 exhibit obvious complex relative permittivity and permeability. In combination with relevant XRD results, it could be concluded that the composition feature determines the naturally electromagnetic properties and the main electromagnetic active component in as-prepared nanocomposites is TiN rather than TiO_2_.

Moreover, T-1000 has much higher *ε*_r_′ and *ε*_r_″ than T-900 in the whole frequency range, and the tan *δ*_E_ value of T-1000 composite is higher than that of T-900 therein. The real relative complex permittivity (*ε*_r_′) and the imaginary part (*ε*_r_″) of relative complex permittivity represent the energy storage ability and loss ability, respectively. Thus, the high imaginary parts of the permittivity is propitious for possessing ideal dielectric loss ability (tan *δ*_*E*_ = *ε*_r_″/*ε*_r_′)^30^. According to the free electron theory, free electrons have distinct effect on the imaginary part of relative complex permittivity and *ε*″ ≈ 1/2 π*ε*_0_ *ρf*, where *ρ* is the resistivity^15^. It could be speculated that the higher *ε*″ values of T-1000 at 2–18 GHz indicate a higher electrical conductivity with respect to T-900 and would result in the increase of dielectric loss. In combination with relevant XRD and Raman data, we can suppose that the improved crystallinity and elevated nitridation degree of TiN obtained at a higher calcining temperature account for the significantly increased conductivity and dielectric loss of T-1000. This is consistent with the work conducted by Vaz *et al.* in that the electrical resistivity of TiN films is mainly determined by its phase composition, microstructure and defects (dislocations, impurities and grain boundaries) and the high quality of TiN results in the increased conductivity^31^.

What should be emphasized is that both T-900 and T-1000 exhibit an anomalous *μ*_r_″ and magnetic loss at 8 ~ 12 GHz, though both TiN and C are not among the traditional magnetic material candidates. Specifically, as-prepared T-900 displays exceptionally high tan *δ*_M_ (its maxima value is 0.60) even over most of conventional magnetic fillers such as carbon nanotubes/Fe_3_O_4_ (~0.34)^32^, zinc ferrite nanofiber (~0.27)^33^, mesoporous Fe_3_O_4_ aerogels (~0.28)^34^, and RGO/Co_3_O_4_ (~0.22)^35^. When compared to traditional microwave absorbing materials such as resistance radar-absorbing materials, dielectric absorbing materials, and magnetic medium absorbent materials, the unexpected high tan *δ*_M_ of TiN/C nanocomposites is unique and worth special attention, which is contributive to improving the consumption of electromagnetic energy and to the coexistence of tan *δ*_E_ and tan *δ*_M_ thereby favoring electromagnetic wave absorption.

**Figure 4:**
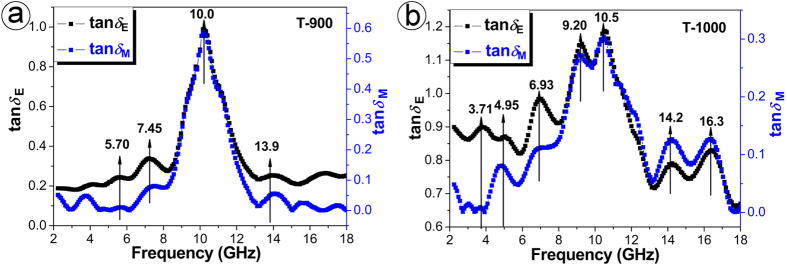
The frequency dependence of dielectric loss (tan *δ*_E_) and magnetic loss (tan *δ*_M_) of paraffin-matrix composites filled with T-900 (**a**) and T-1000 (**b**).

Figure 4 comparatively shows the frequency dependence of tan *δ*_M_ and tan *δ*_E_ of composites T-900 and T-1000. It is seen that the tan *δ*_E_ and tan *δ*_M_ of TiN/C nanocomposites not only vary with varying EM wave frequency in very similar manners, but also exhibit very similar dielectric and magnetic resonance peaks. For example, to T-900, the peaks of dielectric loss overlapped those of magnetic loss at 5.70 GHz, 7.45 GHz, 10.0 GHz, and 13.9 GHz, respectively. To T-1000, the similar resonance electromagnetic peaks could be observed at 3.71 GHz, 4.95 GHz, 6.93 GHz, 9.20 GHz, 10.5 GHz, 14.2 GHz, and 16.3 GHz, respectively. These results confound our traditional understanding of electromagnetism in solids. Because the permittivity and permeability represent two different types of vector states of the electromagnetic wave, and they are connected to the energy stored/consumed in an electric field and a magnetic field, respectively. While considerable efforts have been paid theoretically and experimentally, the similar electromagnetic variation behavior of the permeability and permittivity has not been reported to date. Here, the anomalously electromagnetic resonances imply the similar electric/magnetic energy consumed mechanism and make is possible to achieve an appropriate balance between the permeability and permittivity simultaneously in a simple system.

Further, combining the corresponding Raman spectra of T-900 and T-1000 with their HRTEM images, we can infer that the electromagnetic losses attributed to the naturally physical properties of the absorbing materials and their structures and defects have profound effects on both the magnetic and dielectric loss. For example, the interfacial polarization and the associated relaxation could contribute to the electromagnetic loss, similar to what is observed in other composite system^16^. Besides, various defects can also serve as polarized centers and have a significant effect on the electromagnetic loss. Moreover, under the alternating electromagnetic field, the electrons arising from oxygen vacancies would migrate back and forth in response to both the electric field and the magnetic field, thus leading to lags of polarization and electromagnetic loss. The schematic diagram for possible microwave absorbing mechanism of TiN/C composites is shown in supplementary Fig. S3.

**Figure 5:**
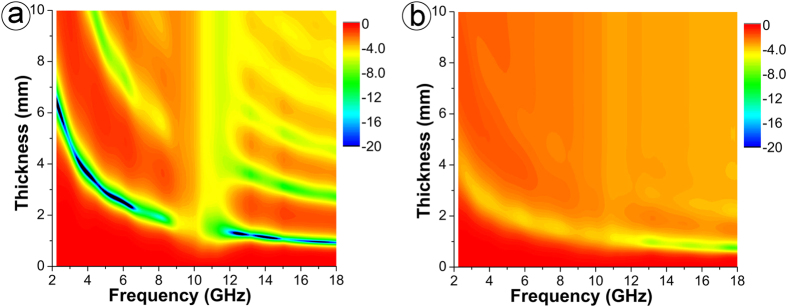
Two-dimensional plots showing frequency dependence of the *RL* of paraffin composites filled with (**a**) T-900 and (**b**) T-1000.

To further prove the dependence of the microwave absorption properties on the permittivity and permeability, we have calculated the reflection losses [*RL* (dB)] of the TiN/C composites according to the transmit line theory^20^. Figure 5 shows the two-dimensional reflection loss mapping plots of the paraffin composites filled with T-900 and T-1000 as functions of frequency and thickness of absorbers. It can be seen that the reflection loss of composite T-1000 is relatively poor (more than −10 dB between 2–18 GHz). This is possibly because composite T-1000 exhibits relatively high dielectric loss and low magnetic loss which counteract the EM matching principle and hinder the absorption of EM wave. In contrast, composite T-900 exhibits enhanced microwave absorption [<−10 dB] in very broad frequency ranges (2.0 ~ 8.8 GHz and 11.7 ~ 18.0 GHz) when its thickness is adjusted from 1 mm to 6 mm. Therefore, it is reasonable to deduce that such a significant enhancement of the microwave wave absorption of composite T-900 is a consequence of the high tan *δ*_M_ and the impedance match between tan *δ*_E_ and tan *δ*_M_.

## Discussion

**Figure 6:**
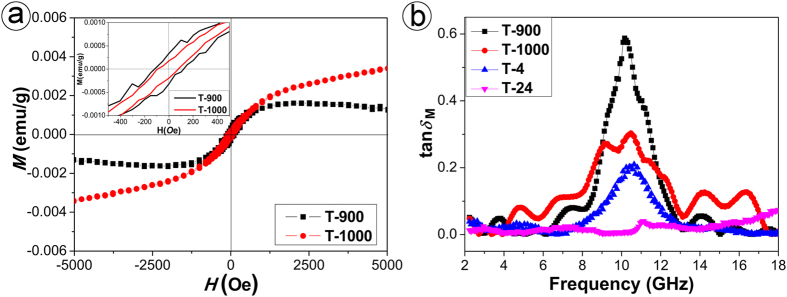
Hysteresis loops of T-900 and T-1000 observed in a range of −5 k*O*e < H < 5 k*O*e at 298 K (**a**) and frequency dependence of magnetic loss (tan δM) of corresponding paraffin composites (**b**), the upper left inset in (**a**) shows the hysteresis loops of the two samples between −500 and 500 *O*e.

To further investigate the relationship between the static and dynamic magnetic properties, we measured the magnetization (*M*) versus applied magnetic field (*H*) curves of T-900 and T-1000 by SQUID at room temperature (298 K). As shown in Fig. 6a, both T-900 and T-1000 possess a distinct ferromagnetic hysteresis loop, corresponding to pronounced room-temperature ferromagnetism. Particularly, a higher nitridation temperature corresponds to an enhanced nitridation degree of NTA as well as an enhanced magnetic moment of as-prepared TiN/C nanocomposite, which is well consistent with what is reported on undoped TiN nanoparticles obtained under different nitriding duration^24^. As to T-900, though it exhibits ferromagnetic behavior with coercive field in a range of −1 k*O*e < *H* < 1 k*O*e, its magnetization at a higher magnetic field decreases in association with a negative slope, which indicates that T-900 exhibits diamagnetic properties^36^. As the magnetization measurement is a bulk effect, the diamagnetic signal might be due to the contributions from the contaminations and a small concentration of impurities below the detecting limits of XRD and Raman or the diamagnetic susceptibility of TiN_1−*x*_O_*x*_^37^. Moreover, we also investigated the dynamic magnetic properties of the paraffin-matrix composites filled with TiN nanoparticles without carbon, T-4 and T-24. To explain the differences between the tan *δ*_M_ of various paraffin-matrix composites filled with T-900, T-1000, T-4 and T-24, we demonstrate the corresponding frequency-tan *δ*_M_ curves in Fig. 6b. Firstly, the tan *δ*_M_ of the paraffin-matrix composites separately filled with T-4, T-900 and T-1000 changes with the frequency in similar manner, which might be governed by their similar composition feature and microstructure defects. Secondly, sample T-900 exhibits a larger dynamic permeability than un-doped TiN (T-4). Considering that the only difference between samples T-900 and T-4 is that the latter is obtained under similar conditions without carbon, we can stipulate that the incorporation of carbon is responsible for the improvement in the magnetic loss of TiN nanocrystallites. Thirdly, elevating nitridation degree corresponds to a decrease in the dynamic permeability of various samples (comparison between T-4 and T-24, or between T-900 and T-1000). Based on Snoek theory (μ*f_*r*_ ∝ *M*_*s*_), the permeability and the resonance frequency are proportional to the saturation magnetization^38^. Evidently, this theory is invalid in the present research and there is no straightforward dependence between the static magnetic properties and the dynamic magnetic properties of TiN/C nanocomposites. On the one hand, the evident microwave dynamic permeability could not be attributed to their weak static magnetic properties. On the other hand, the static and dynamic magnetic properties show the opposite trend, increasing nitridation degree refers to an enhanced static magnetic moment but a decreased dynamic permeability. Hopefully, this work could enlighten us to understand the intrinsic reasons for microwave absorption mechanism and to develop new absorbing materials with excellent microwave absorption performances. However, it should be noted that the exact mechanism is relatively complicated and requires further theoretical analysis.

## Conclusion

Herein, we adopt titanium nitride-carbon (TiN/C) nanocomposite as a concrete example to investigate both the static and dynamic magnetic behaviors in relation to their structural defects. The results indicate that governed by the composition feature and microstructural defects, TiN/C nanocomposites exhibit not only distinct static magnetic properties but also exceptionally high dynamic permeability. Besides, TiN/C nanocomposites exhibit anomalously electromagnetic resonances, which means that there are some similar mechanisms between the permeability and permittivity. This is challenging for the traditional understanding of electromagnetism and makes it possible to achieve an appropriate balance between the permeability and permittivity simultaneously in a simple system. Particularly, sample T-900 has a lower magnetic moment but a higher dynamic permeability than T-1000, which is contradictive against classical physics theory in that a higher dynamic permeability refers to a higher magnetism and means there is no straightforward dependence between the weak static magnetic properties and the dynamic magnetic properties of TiN/C nanocomposites. This discovery gives a typical example to renew the unique electromagnetic characteristics of nanomaterials. Further theoretical and experimental efforts are also needed to better recognize the interactions between the electromagnetism and structural defects (the type and content of structural defects, or even their location and physical properties) of the title nanostructures and other non-magnetic nanostructures. This work, hopefully, is not only to provide some valuable clues to exploring the magnetism and absorbing mechanism of nanostructures but also to open up a novel and effective avenue to modulate the electromagnetic properties of MAM.

## Methods

### Synthesis of TiN/C nanocomposites

TiN/C nanocomposites were prepared with nanotubular titanic acid (H_2_Ti_2_O_4_(OH)_2_, denoted as NTA) and polyacrylamide (denoted as PAM) as the starting materials. Briefly, 1.90 g of NTA powder (the preparation of NTA is described elsewhere^25^) was dispersed in 50 mL of deionized water with the help of ultrasonic oscillation. Then 2.84 g of PAM was added into the solution and stirred vigorously until a uniform viscous dispersion was obtained. Resultant viscous dispersion was dried under vacuum at 80 °C to obtain a fluffy precursor (denoted as NTA/PAM). As-obtained NTA/PAM precursor was nitrided for 4 h at 400 °C, 700 °C, 900 °C, and 1000 °C, respectively. Corresponding nitrided products are denoted as T-400, T-700, T-900, and T-1000, respectively. Moreover, TiN nanoparticles were also prepared for comparative studies under the same conditions while no carbon was introduced. Corresponding products without carbon, obtained by nitriding NTA at 900 °C for 4 h and 24 h, respectively, are denoted as T-4 and T-24.

### Characterization techniques

The morphology of as-prepared products was examined with a transmission electron microscope (TEM, JEOL JEM-2010; accelerating voltage 200 kV), and a Philips X’Pert Pro X-ray diffractometer (XRD) was performed to analyze their phase structure (Cu-*K*α radiation, *λ* = 1.5406 Å). Raman scattering spectra of as-prepared products were recorded with a RM-1000 facility (Renishaw; excitation source: 632.8 nm laser). Magnetic measurements were conducted with a superconducting quantum interference device (SQUID, MPMS XL-7, Quantum Design).

### Electromagnetic parameter measurements

A proper amount of as-synthesized products was homogeneously dispersed in paraffin (paraffin as a binder is transparent for electromagnetic wave) at a mass fraction of 45:55 and then pressed into a toroidally-shaped sample with an inner diameter of 3.04 mm and an outer diameter of 7.00 mm for measurements of electromagnetic properties. The real and imaginary parts of the complex permittivity and permeability of resultant paraffin-based composites were measured in a frequency range of 2 ~ 18 GHz with an Agilent N5230A vector network analyzer. The reflection loss, *RL* (*dB*), is an effective evaluation standard of the microwave absorbance capacity of metal-backed slabs of material, and low reflection corresponds to high absorption. It has been proven that the directly-measured and calculated *RL* are in remarkable agreement, due to the same underlying physical origin. To investigate the application potential of as-fabricated TiN/C composites, we also calculated their frequency dependence *RL* (*dB*) values according to the transmit-line theory by using the measured data of relative complex permeability and permittivity^25^.

## Additional Information

**How to cite this article**: Gong, C. *et al.* Unique Static Magnetic and Dynamic Electromagnetic Behaviors in Titanium Nitride/Carbon Composites Driven by Defect Engineering. *Sci. Rep.* **6**, 18927; doi: 10.1038/srep18927 (2016).

## Supplementary Material

Supplementary Information
