# Supplementary material for: Unique Static Magnetic and Dynamic Electromagnetic Behaviors in Titanium Nitride/Carbon Composites Driven by Defect Engineering
Source: Sci Rep. 2016 Jan 7;6:18927. doi: 10.1038/srep18927 (PMC4703962; doi:10.1038/srep18927)
Supplement: Supplementary Information [file srep18927-s1.pdf]

Unique Static Magnetic and Dynamic Electromagnetic Behaviors in Titanium Nitride/Carbon Composites Driven by Defect Engineering

Chunhong Gong[1], Hongjie Meng [1,2], Xiaowei Zhao[1], Xuefeng Zhang[3], Laigui Yu[2], Jingwei Zhang[2*] & Zhijun Zhang[2]

[1]College of Chemistry and Chemical Engineering, Henan University, Kaifeng 475004, P. R.China

[2] Engineering Research Center for Nanomaterials, Henan University, Kaifeng 475004, P. R. China

[3]Key Laboratory for Anisotropy and Texture of Materials, Northeastern University, Shenyang 110819, P. R. China

*corresponding author: jwzhang@henu.edu.cn

Supplementary Informations

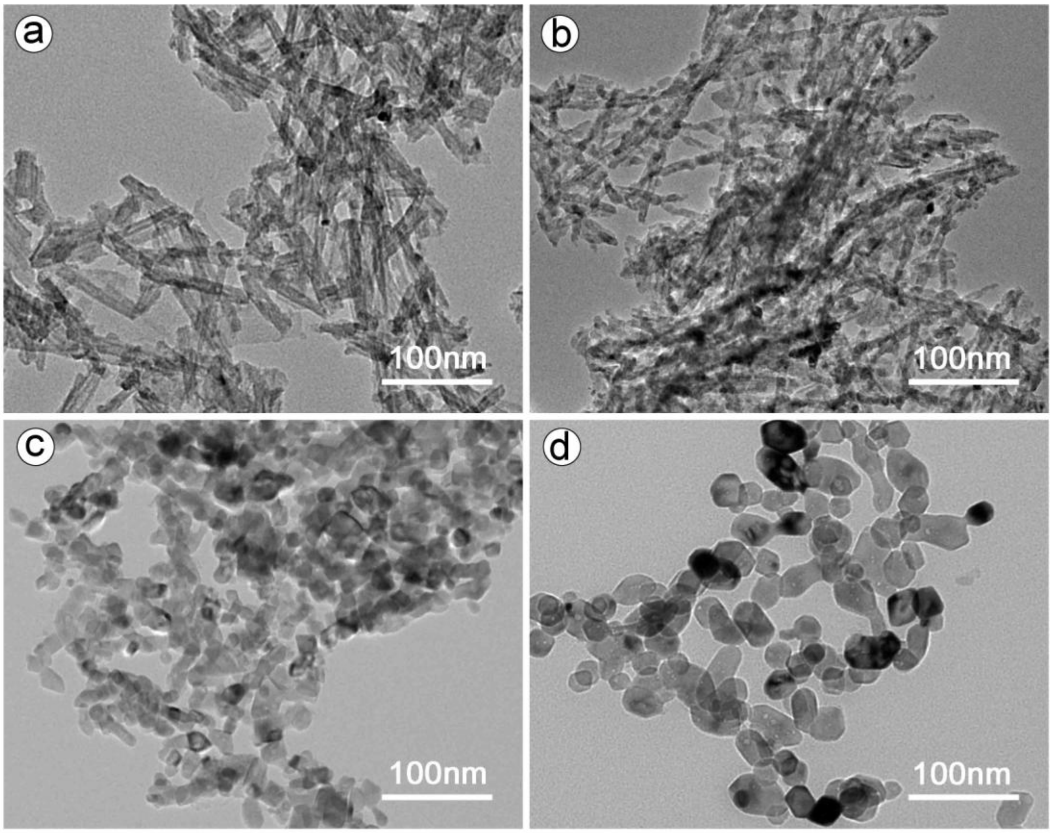

Figure S1. TEM images of T-400 (a), T-700 (b), T-900 (c), and T-1000 (d).

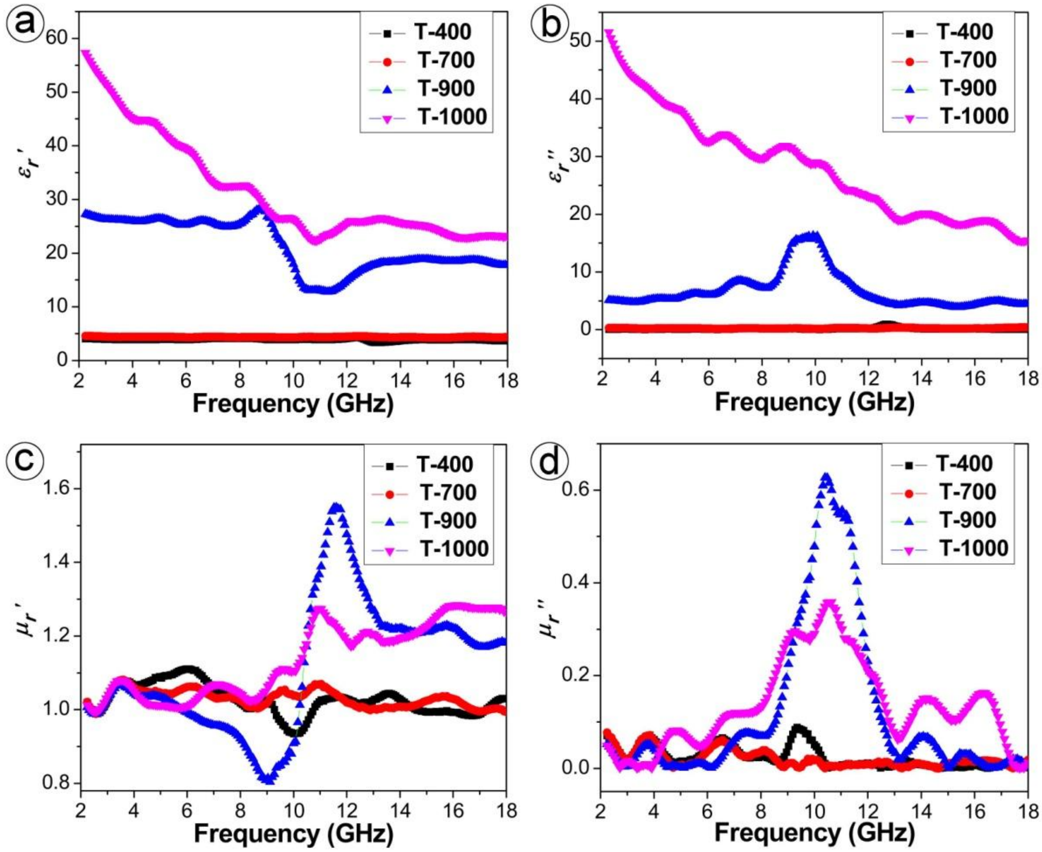

Figure S2. The frequency dependence of the complex relative permittivity and permeability of the paraffin composites filled with T-400, T-700, T-900 and T-1000.

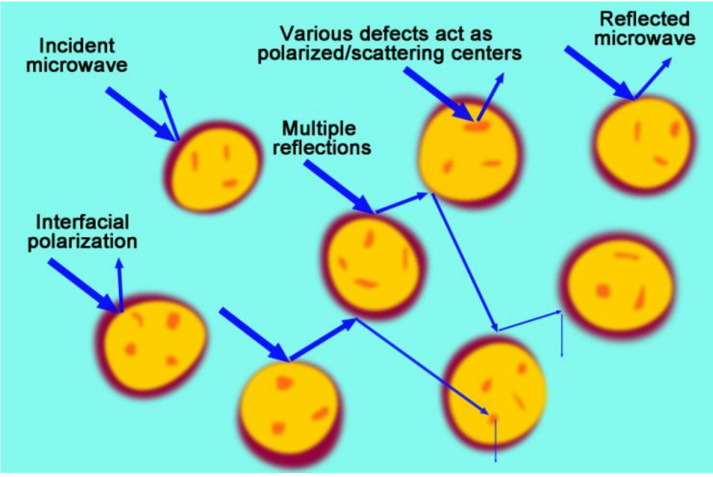

Figure S3. The schematic diagram for the possible microwave absorbing mechanism of TiN/C composites.
